# Supplementary material for: Missense mutations in intrinsically disordered protein regions link pathogenicity and phase separation
Source: J Biol Chem. 2025 Sep 29;301(11):110773. doi: 10.1016/j.jbc.2025.110773 (PMC12597276; doi:10.1016/j.jbc.2025.110773)
Supplement: Supporting information [file mmc1.docx]

**Supporting Information for:**

**Missense mutations in intrinsically disordered protein regions link pathogenicity and phase separation**

Short title: Missense mutations in phase-separating IDRs

*Oliver L. Kipp, Karen A. Lewis, Loren E. Hough, and Steven T. Whitten*

**Contents:**

Supporting Tables

S1. Human proteome, missense mutation, SLiM, and phosphorylation statistics.

S2. Normalized missense mutation rate calculations.

S3. AUC and *p*-value for protein sets annotated as disease associated in UniProt.

Supporting Figures

S1. The ParSe algorithm (version 2).

S2. Predicted PS potential in the reference human proteome and homotypic PS proteins.

S3. Enrichment for phase separation in protein sets.

S4. Protein length and percent of residues folded in PS and nonPS proteins.

S5. Pathogenic versus benign odds ratios for missense mutations.

S6. Pathogenic missense mutation rate by region class for the natural variants index.

S7. Pathogenic missense mutation rate increases in SLiMs by region class.

S8. Quantifying differences in predicted PS potential between protein sets.

S9. AUC trends with enrichment for phase separation behavior.

S10. AUC rank order for protein sets annotated as disease associated.

S11. Frequency of single amino acid missense variation types.

S12. Frequency of single amino acid missense variation types in PS IDRs.

S13. Pathogenic minus benign difference frequencies for single amino acid missense variation types in nonPS and folded regions.

S14. Predicting clinical significance of missense mutations in the natural variants index.

Supporting References

**Supporting Tables**

**Table S1. Human proteome, missense mutation, SLiM, and phosphorylation statistics.**

proteins *^a^* 20,435

proteins w/ *N*≥25 20,383

PS proteins w/ *N*≥25 3,338

nonPS proteins w/ *N*≥25 17,045

residues 11,403,806

residues in PS proteins 3,810,609

residues in nonPS proteins 7,593,197

residues in PS IDRs 624,550

residues in nonPS IDRs 1,061,254

residues in folded regions 8,378,569

pathogenic mutations *^b^* 32,665

pathogenic mutations in PS proteins 9,083

pathogenic mutations in nonPS proteins 23,582

pathogenic mutations in PS IDRs 1,186

pathogenic mutations in nonPS IDRs 727

pathogenic mutations in folded regions 29,225

proteins with pathogenic mutations 3,345

PS proteins with pathogenic mutations 706

nonPS proteins with pathogenic mutations 2,639

benign mutations 39,654

benign mutations in PS proteins 10,995

benign mutations in nonPS proteins 28,659

benign mutations in PS IDRs 2,191

benign mutations in nonPS IDRs 3,828

benign mutations in folded regions 28,812

proteins with benign mutations 11,681

PS proteins with benign mutations 2,301

nonPS proteins with benign mutations 9,380

uncertain clinical significance mutations 10,177

uncertain clinical significance mutations in PS proteins 3,361

uncertain clinical significance mutations in nonPS proteins 6,816

uncertain clinical significance mutations in PS IDRs 335

uncertain clinical significance mutations in nonPS IDRs 780

uncertain clinical significance mutations in folded regions 8,149

proteins with uncertain clinical significance mutations 2,802

PS proteins with uncertain clinical significance mutations 743

nonPS proteins with uncertain clinical significance mutations 2,059

SLiMs *^c^* 2,247

proteins with a SLiM 1,370

PS proteins with a SLiM 523

nonPS proteins with a SLiM 847

SLiM residues 16,224

SLiMs in PS proteins 964

SLiMs in nonPS proteins 1,282

SLiMs in PS IDRs 222

SLiM residues in PS IDRs 1,410

SLiMs in nonPS IDRs 533

SLiM residues in nonPS IDRs 3,668

SLiMs in folded regions 692

SLiM residues in folded regions 5,834

pathogenic mutations in SLiMs 141

pathogenic mutations in SLiMs in PS IDRs 44

pathogenic mutations in SLiMs in nonPS IDRs 18

pathogenic mutations in SLiMs in folded regions 52

benign mutations in SLiMs 76

benign mutations in SLiMs in PS IDRs 8

benign mutations in SLiMs in nonPS IDRs 11

benign mutations in SLiMs in folded regions 35

uncertain clinical significance mutations in SLiMs 194

uncertain clinical significance mutations in SLiMs in PS IDRs 23

uncertain clinical significance mutations in SLiMs in nonPS IDRs 31

uncertain clinical significance mutations in SLiMs in folded regions 32

phosphorylation sites *^d^* 531,023

proteins with a phosphorylation site 30,600

PS proteins with a phosphorylation site 5,763

nonPS proteins with a phosphorylation site 24,837

phosphorylation sites in PS proteins 212,256

phosphorylation sites in nonPS proteins 318,767

phosphorylation sites in PS IDRs 79,280

phosphorylation sites in nonPS IDRs 87,645

phosphorylation sites in folded regions 263,030

pathogenic mutations at phosphorylation sites 942

pathogenic mutations at phosphorylation sites in PS IDRs 54

pathogenic mutations at phosphorylation sites in nonPS IDRs 58

pathogenic mutations at phosphorylation sites in folded regions 746

benign mutations at phosphorylation sites 1,542

benign mutations at phosphorylation sites in PS IDRs 210

benign mutations at phosphorylation sites in nonPS IDRs 256

benign mutations at phosphorylation sites in folded regions 746

uncertain clinical significance mutations at phosphorylation sites 403

uncertain clinical significance mutations at phosphorylation sites in PS IDRs 45

uncertain clinical significance mutations at phosphorylation sites in nonPS IDRs 51

uncertain clinical significance mutations at phosphorylation sites in folded regions 239

*^a^* Human protein sequences were obtained from the UniProt reference human proteome (1). For a protein, ParSe analysis (2) requires a minimum sequence length, *N*, of 25 residues. The table statistics, other than the first row, thus includes contributions only from proteins with *N*≥25.

*^b^* Mutant sites represent the single amino acid missense mutations in the humsavar index (1).

*^c^* SLiMs sites in human proteins were obtained from the Eukaryotic Linear Motif resource (3).

*^d^* Phosphorylation sites in human proteins were obtained from the Eukaryotic Phosphorylation Site Database 2.0 (4).

**Table S2. Normalized missense mutation rate calculations.** Input values from Table S1.

pathogenic mutations in folded regions = (29,225/32,665)/(8,378,569/11,403,806) = 1.218

pathogenic mutations in PS IDRs = (1,186/32,665)/(624,550/11,403,806) = 0.663

pathogenic mutations in nonPS IDRs = (727/32,665)/(1,061,254/11,403,806) = 0.239

benign mutations in folded regions = (28,812/39,654)/(8,378,569/11,403,806) = 0.989

benign mutations in PS IDRs = (2,191/39,654)/(624,550/11,403,806) = 1.009

benign mutations in nonPS IDRs = (3,828/39,654)/(1,061,254/11,403,806) = 1.037

SLiMs in folded regions = (692/2,247)/(8,378,569/11,403,806) = 0.419

SLiMs in PS IDRs = (222/2,247)/(624,550/11,403,806) = 1.804

SLiMs in nonPS IDRs = (533/2,247)/(1,061,254/11,403,806) = 2.549

phosphorylation sites in folded regions = (263,030/531,023)/(8,378,569/11,403,806) = 0.674

phosphorylation sites in PS IDRs = (79,280/531,023)/(624,550/11,403,806) = 2.726

phosphorylation sites in nonPS IDRs = (87,645/531,023)/(1,061,254/11,403,806) = 1.774

pathogenic mutations in SLiMs = (141/32,665)/(16,224/11,403,806) = 3.034

pathogenic mutations in SLiMs in folded regions = (52/32,665)/(5,834/11,403,806) = 3.112

pathogenic mutants in SLiMs in PS IDRs = (44/32,665)/(1,410/11,403,806) = 10.894

pathogenic mutants in SLiMs in nonPS IDRs = (18/32,665)/(3,668/11,403,806) = 1.713

benign mutations in SLiMs = (76/39,654)/(16,224/11,403,806) = 1.347

benign mutations in SLiMs in folded regions = (35/39,654)/(5,834/11,403,806) = 1.725

benign mutations in SLiMs in PS IDRs = (8/39,654)/(1,410/11,403,806) = 1.632

benign mutations in SLiMs in nonPS IDRs = (11/39,654)/(3,668/11,403,806) = 0.862

pathogenic mutations at phosphorylation sites = (942/32,665)/(531,023/11,403,806) = 0.619

pathogenic mutations at phosphorylation sites in folded regions = (746/32,665)/(263,030/11,403,806) = 0.990

pathogenic mutations at phosphorylation sites in PS IDRs = (54/32,665)/(79,280/11,403,806) = 0.238

pathogenic mutations at phosphorylation sites in nonPS IDRs = (58/32,665)/(87,645/11,403,806) = 0.231

benign mutations at phosphorylation sites = (1,542/39,654)/(531,023/11,403,806) = 0.835

benign mutations at phosphorylation sites in folded regions = (746/39,654)/(263,030/11,403,806) = 0.816

benign mutations at phosphorylation sites in PS IDRs = (210/39,654)/(79,280/11,403,806) = 0.762

benign mutations at phosphorylation sites in nonPS IDRs = (256/39,654)/(87,645/11,403,806) = 0.840

**Table S3. AUC and *p*-value for protein sets annotated as disease associated in UniProt.**

| **Set*^a^*** | **Proteins*^b^*** | **PS proteins*^c^*** | **AUC*^d^*** | **mean AUC*^e^*** | **𝜎*^f^*** | **(AUC - mean AUC)/𝜎** | ***p*-value*^g^*** |
| --- | --- | --- | --- | --- | --- | --- | --- |
| All | 4638 | 1046 | 0.5627 | 0.5004 | 0.0037 | 16.7677 | < 2.2E-16 |
| Disease variant | 3756 | 817 | 0.5575 | 0.4994 | 0.0036 | 16.0386 | < 2.2E-16 |
| Intellectual disability | 703 | 238 | 0.6286 | 0.4986 | 0.0104 | 12.4926 | < 2.2E-16 |
| Proto-oncogene | 231 | 86 | 0.6482 | 0.4997 | 0.0188 | 7.9015 | 4.31E-15 |
| Deafness | 285 | 73 | 0.5961 | 0.4981 | 0.0156 | 6.2994 | 1.75E-08 |
| Epilepsy | 297 | 75 | 0.5939 | 0.4981 | 0.0156 | 6.1580 | 1.98E-08 |
| Autism spectrum disorder | 65 | 33 | 0.7268 | 0.5011 | 0.0397 | 5.6793 | 3.06E-10 |
| Tumor suppressor | 183 | 56 | 0.6103 | 0.5015 | 0.0194 | 5.5967 | 2.14E-07 |
| Neurodegeneration | 413 | 79 | 0.5725 | 0.4989 | 0.0136 | 5.3997 | 3.73E-07 |
| Epidermolysis bullosa | 16 | 10 | 0.8623 | 0.4904 | 0.0704 | 5.2796 | 5.27E-07 |
| Dwarfism | 211 | 58 | 0.5948 | 0.5013 | 0.0201 | 4.6403 | 1.14E-06 |
| Primary microcephaly | 37 | 16 | 0.6937 | 0.4958 | 0.0444 | 4.4625 | 4.33E-05 |
| Holoprosencephaly | 11 | 9 | 0.8949 | 0.5002 | 0.0905 | 4.3617 | 5.50E-06 |
| Craniosynostosis | 26 | 10 | 0.7226 | 0.5031 | 0.0601 | 3.6542 | 8.55E-05 |
| Palmoplantar keratoderma | 39 | 16 | 0.6514 | 0.4978 | 0.0426 | 3.6020 | 0.0008523 |
| Stickler syndrome | 7 | 6 | 0.9681 | 0.5048 | 0.1304 | 3.5529 | 1.73E-05 |
| Ectodermal dysplasia | 55 | 15 | 0.6356 | 0.4969 | 0.0404 | 3.4356 | 0.0004134 |
| Ehlers-Danlos syndrome | 17 | 7 | 0.6907 | 0.5000 | 0.0597 | 3.1951 | 0.006468 |
| Atrial septal defect | 8 | 5 | 0.8126 | 0.4936 | 0.1008 | 3.1646 | 0.002136 |
| Congenital hypothyroidism | 19 | 9 | 0.6873 | 0.4949 | 0.0610 | 3.1548 | 0.003914 |
| Hypotrichosis | 40 | 15 | 0.6248 | 0.4977 | 0.0408 | 3.1126 | 0.005988 |
| Cone-rod dystrophy | 30 | 8 | 0.6366 | 0.4930 | 0.0496 | 2.8928 | 0.009102 |
| Neuropathy | 129 | 24 | 0.5716 | 0.4994 | 0.0260 | 2.7765 | 0.004794 |
| Premature ovarian failure | 22 | 7 | 0.6604 | 0.5026 | 0.0581 | 2.7178 | 0.008063 |
| Diabetes mellitus | 86 | 22 | 0.5879 | 0.4998 | 0.0331 | 2.6621 | 0.004732 |
| Corneal dystrophy | 21 | 6 | 0.6409 | 0.4883 | 0.0602 | 2.5370 | 0.02663 |
| Alport syndrome | 7 | 4 | 0.8337 | 0.5048 | 0.1304 | 2.5222 | 0.002096 |
| Retinitis pigmentosa | 108 | 25 | 0.5720 | 0.4985 | 0.0293 | 2.5094 | 0.007915 |
| Nephronophthisis | 22 | 6 | 0.6416 | 0.5026 | 0.0581 | 2.3941 | 0.02336 |
| Cardiomyopathy | 102 | 27 | 0.5652 | 0.5008 | 0.0282 | 2.2847 | 0.02953 |
| Age-related macular degeneration | 16 | 5 | 0.6463 | 0.4904 | 0.0704 | 2.2130 | 0.04373 |
| Microphthalmia | 30 | 8 | 0.5963 | 0.4930 | 0.0496 | 2.0810 | 0.05867 |
| Kallmann syndrome | 21 | 3 | 0.6049 | 0.4883 | 0.0602 | 1.9387 | 0.09627 |
| Oncogene | 12 | 2 | 0.6426 | 0.4965 | 0.0760 | 1.9229 | 0.08336 |
| Thrombophilia | 11 | 4 | 0.6696 | 0.5002 | 0.0905 | 1.8718 | 0.04845 |
| Parkinsonism | 41 | 9 | 0.5863 | 0.4987 | 0.0470 | 1.8634 | 0.05735 |
| Schizophrenia | 19 | 7 | 0.6080 | 0.4949 | 0.0610 | 1.8546 | 0.106 |
| Myofibrillar myopathy | 13 | 5 | 0.6355 | 0.4928 | 0.0777 | 1.8375 | 0.09324 |
| Peters anomaly | 5 | 3 | 0.7007 | 0.4879 | 0.1202 | 1.7706 | 0.1244 |
| SCID | 20 | 3 | 0.6099 | 0.4989 | 0.0629 | 1.7634 | 0.08259 |
| Amelogenesis imperfecta | 25 | 4 | 0.5984 | 0.5071 | 0.0534 | 1.7109 | 0.08422 |
| Hemolytic uremic syndrome | 10 | 2 | 0.6684 | 0.5049 | 0.0956 | 1.7095 | 0.06941 |
| Hypogonadotropic hypogonadism | 33 | 5 | 0.5841 | 0.4931 | 0.0538 | 1.6909 | 0.09757 |
| Hereditary nonpolyposis colorectal cancer | 8 | 1 | 0.6570 | 0.4936 | 0.1008 | 1.6212 | 0.1243 |
| Emery-Dreifuss muscular dystrophy | 6 | 3 | 0.6880 | 0.5098 | 0.1144 | 1.5573 | 0.1042 |
| Ciliopathy | 168 | 27 | 0.5279 | 0.4997 | 0.0192 | 1.4734 | 0.229 |
| Parkinson disease | 24 | 5 | 0.5915 | 0.4992 | 0.0670 | 1.3778 | 0.1168 |
| Congenital stationary night blindness | 14 | 4 | 0.5951 | 0.5040 | 0.0695 | 1.3107 | 0.1922 |
| Glaucoma | 14 | 3 | 0.5946 | 0.5040 | 0.0695 | 1.3035 | 0.2039 |
| Systemic lupus erythematosus | 22 | 4 | 0.5729 | 0.5026 | 0.0581 | 1.2112 | 0.2298 |
| Cockayne syndrome | 6 | 2 | 0.6462 | 0.5098 | 0.1144 | 1.1920 | 0.2089 |
| Hereditary hemolytic anemia | 40 | 6 | 0.5434 | 0.4977 | 0.0408 | 1.1198 | 0.328 |
| Xeroderma pigmentosum | 9 | 2 | 0.5729 | 0.4886 | 0.0858 | 0.9832 | 0.3938 |
| Alzheimer disease | 18 | 3 | 0.5768 | 0.5056 | 0.0728 | 0.9775 | 0.256 |
| Albinism | 21 | 1 | 0.5461 | 0.4883 | 0.0602 | 0.9614 | 0.4661 |
| Asthma | 12 | 1 | 0.5635 | 0.4965 | 0.0760 | 0.8820 | 0.4253 |
| Hirschsprung disease | 10 | 4 | 0.5876 | 0.5049 | 0.0956 | 0.8646 | 0.379 |
| Osteogenesis imperfecta | 26 | 7 | 0.5549 | 0.5031 | 0.0601 | 0.8629 | 0.2869 |
| Williams-Beuren syndrome | 29 | 6 | 0.5384 | 0.4926 | 0.0559 | 0.8206 | 0.4594 |
| Long QT syndrome | 16 | 5 | 0.5468 | 0.4904 | 0.0704 | 0.8004 | 0.4959 |
| Aortic aneurysm | 16 | 4 | 0.5445 | 0.4904 | 0.0704 | 0.7677 | 0.5055 |
| Glycogen storage disease | 22 | 2 | 0.5468 | 0.5026 | 0.0581 | 0.7618 | 0.4351 |
| Lissencephaly | 34 | 8 | 0.5358 | 0.5004 | 0.0475 | 0.7435 | 0.4132 |
| Brugada syndrome | 10 | 4 | 0.5759 | 0.5049 | 0.0956 | 0.7423 | 0.3917 |
| Mucopolysaccharidosis | 14 | 0 | 0.5521 | 0.5040 | 0.0695 | 0.6917 | 0.507 |
| Congenital myasthenic syndrome | 25 | 6 | 0.5435 | 0.5071 | 0.0534 | 0.6822 | 0.4091 |
| Atrial fibrillation | 15 | 4 | 0.5396 | 0.4885 | 0.0794 | 0.6437 | 0.5408 |
| Limb-girdle muscular dystrophy | 40 | 11 | 0.5214 | 0.4977 | 0.0408 | 0.5812 | 0.6741 |
| Atherosclerosis | 8 | 3 | 0.5478 | 0.4936 | 0.1008 | 0.5381 | 0.6379 |
| Glutaricaciduria | 5 | 0 | 0.5470 | 0.4879 | 0.1202 | 0.4916 | 0.7078 |
| Primary hypomagnesemia | 8 | 2 | 0.5384 | 0.4936 | 0.1008 | 0.4448 | 0.7366 |
| Osteopetrosis | 11 | 2 | 0.5362 | 0.5002 | 0.0905 | 0.3976 | 0.6242 |
| Obesity | 66 | 9 | 0.5144 | 0.5011 | 0.0397 | 0.3346 | 0.7245 |
| Cushing syndrome | 11 | 1 | 0.5280 | 0.5002 | 0.0905 | 0.3069 | 0.6752 |
| Amyloidosis | 31 | 4 | 0.5148 | 0.4997 | 0.0538 | 0.2813 | 0.8068 |
| Leber congenital amaurosis | 27 | 3 | 0.5102 | 0.4980 | 0.0503 | 0.2421 | 0.8552 |
| Intrahepatic cholestasis | 18 | 3 | 0.5221 | 0.5056 | 0.0728 | 0.2262 | 0.6838 |
| Aicardi-Goutieres syndrome | 8 | 0 | 0.5044 | 0.4936 | 0.1008 | 0.1076 | 0.9445 |
| Bartter syndrome | 6 | 0 | 0.5149 | 0.5098 | 0.1144 | 0.0446 | 0.9116 |
| Congenital erythrocytosis | 6 | 2 | 0.5094 | 0.5098 | 0.1144 | -0.0035 | 0.9408 |
| Dystonia | 45 | 6 | 0.4985 | 0.4996 | 0.0451 | -0.0235 | 0.9486 |
| Pseudohermaphroditism | 7 | 2 | 0.4998 | 0.5048 | 0.1304 | -0.0382 | 0.9221 |
| Peroxisome biogenesis disorder | 15 | 0 | 0.4806 | 0.4885 | 0.0794 | -0.0990 | 0.8703 |
| Dyskeratosis congenita | 13 | 1 | 0.4760 | 0.4928 | 0.0777 | -0.2165 | 0.8201 |
| Allergen | 6 | 1 | 0.4739 | 0.5098 | 0.1144 | -0.3137 | 0.7693 |
| Leukodystrophy | 45 | 2 | 0.4834 | 0.4996 | 0.0451 | -0.3583 | 0.7107 |
| Cataract | 96 | 11 | 0.4842 | 0.4964 | 0.0293 | -0.4176 | 0.668 |
| Ichthyosis | 49 | 10 | 0.4802 | 0.4997 | 0.0398 | -0.4912 | 0.8082 |
| Gangliosidosis | 5 | 0 | 0.4095 | 0.4879 | 0.1202 | -0.6525 | 0.4818 |
| Fanconi anemia | 21 | 5 | 0.4487 | 0.4883 | 0.0602 | -0.6574 | 0.5252 |
| Heterotaxy | 14 | 2 | 0.4567 | 0.5040 | 0.0695 | -0.6815 | 0.6306 |
| Congenital muscular dystrophy | 30 | 6 | 0.4582 | 0.4930 | 0.0496 | -0.7009 | 0.4323 |
| Diabetes insipidus | 4 | 0 | 0.4158 | 0.5281 | 0.1514 | -0.7415 | 0.5775 |
| Nemaline myopathy | 11 | 2 | 0.3698 | 0.5002 | 0.0905 | -1.4414 | 0.1357 |
| Congenital adrenal hyperplasia | 6 | 0 | 0.2572 | 0.5098 | 0.1144 | -2.2074 | 0.04577 |
| Congenital generalized lipodystrophy | 5 | 0 | 0.2214 | 0.4879 | 0.1202 | -2.2178 | 0.04105 |
| Dystroglycanopathy | 18 | 0 | 0.3246 | 0.5056 | 0.0728 | -2.4867 | 0.009927 |
| Chronic granulomatous disease | 6 | 0 | 0.2246 | 0.5098 | 0.1144 | -2.4923 | 0.01442 |
| Congenital disorder of glycosylation | 53 | 2 | 0.3737 | 0.5014 | 0.0401 | -3.1869 | 0.001243 |
| Diamond-Blackfan anemia | 20 | 0 | 0.1824 | 0.4989 | 0.0629 | -5.0293 | 2.49E-06 |
| Primary mitochondrial disease | 197 | 1 | 0.3227 | 0.4982 | 0.0217 | -8.0974 | < 2.2E-16 |

*^a^* Disease-associated human proteins were annotated and grouped by UniProt (1). “All” refers to the full set of disease-associated proteins and “Disease variant” is the set containing all proteins for which at least one genetic variant involved in a disease has been reported.

*^b^* Number of proteins in the set with sequence length at least 25 residues, the minimum sequence length required for ParSe analysis (2).

*^c^* Number of ParSe-predicted PS proteins in the set.

*^d^* AUC for the set using the reference human proteome as the comparison set.

*^e^* AUC mean from one hundred random human protein sets using the set size given in column 2.

*^f^* AUC standard deviation from one hundred random human protein sets using the set size given in column 2.

*^g^* One-tail *p*-value determined by the Mann-Whitney *U*-test (5) for the calculated PS potentials of the set and using the reference human proteome for the comparison set.

**Supporting Figures**


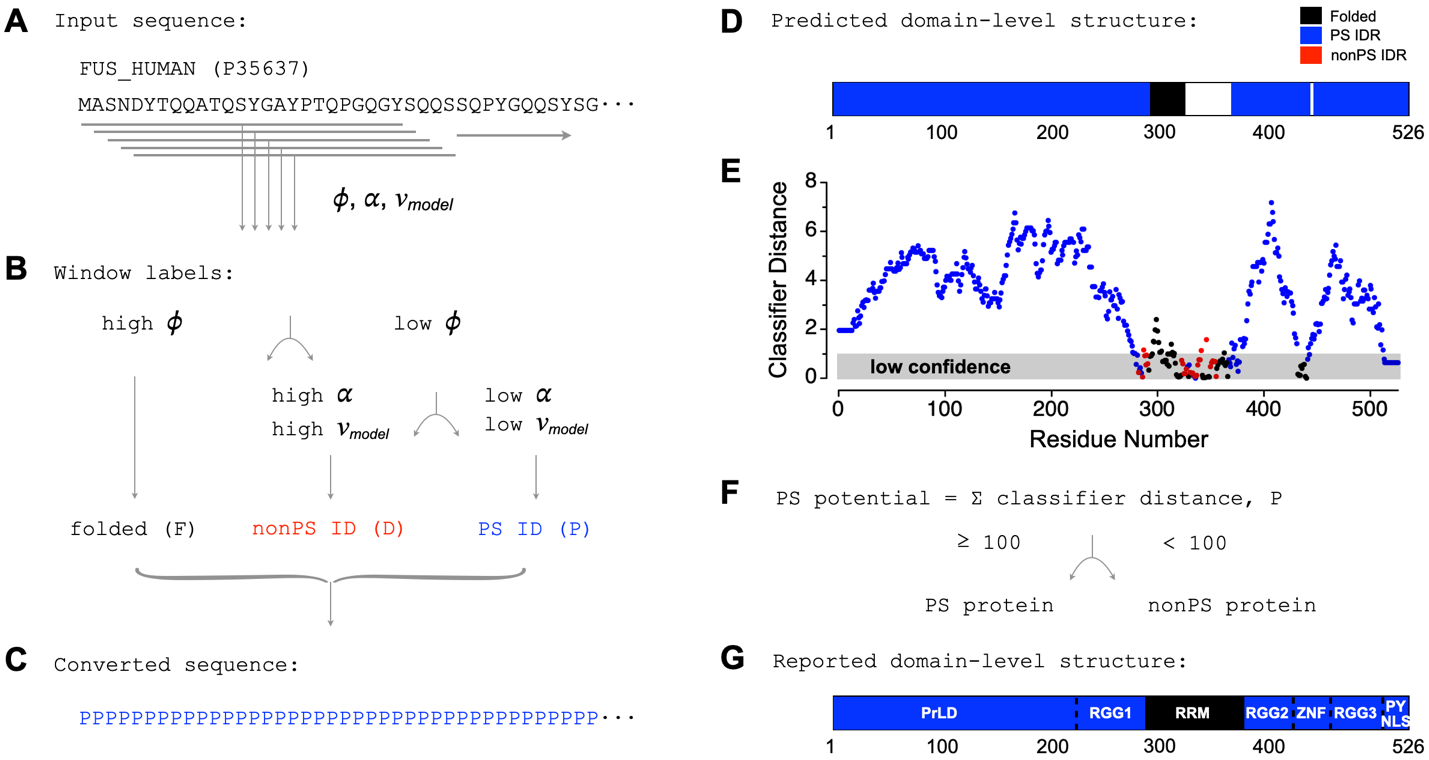


**Figure S1. The ParSe algorithm (version 2).** (**A**) Hydrophobicity (*ϕ*), α-helix propensity (*α*), and *v_model_* are calculated for each contiguous stretch of 25-residues, or “window”, in the primary sequence. (**B**) Each window is assigned a label, F, D, or P, depending on the values of *ϕ*, *α*, and *v_model_*. (**C**) Window labels are assigned to the central residue of the window. Terminal residues are assigned labels according to the first and last windows. (**D**) Contiguous regions of at least 20 residues that are 90% of only one label P, D, or F are colored blue, red, or black, respectively, to represent predicted PS IDRs, IDRs, or folded regions. White corresponds to regions with mixed P, D, or F labels. (**E**) Classifier distance of each window, assigned to the central residue, and colored according to its label P (blue), D (red), or F (black). This “distance” represents standard deviations from the high/low cutoff values of *ϕ*, *α*, and *v_model_*, where standard deviations were determined from the distributions of *ϕ*, *α*, and *v_model_* in the sequences that trained ParSe v2. Low values (<1) indicate low statistical confidence for a window label. (**F**) The classifier distance sum from P-labeled windows is used as a “PS potential”; primary sequences with values ≥100 are PS predicted proteins. (**G**) Reported domain-level organization for the FUS RNA-binding protein (6, 7).


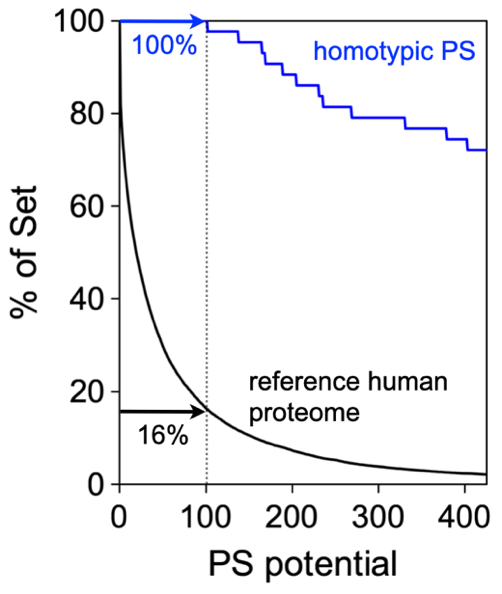


**Figure S2.** **Predicted PS potential in the reference human proteome and homotypic PS proteins.** The percent of proteins in a set with sequence-calculated PS potential equal to or greater than the value indicated by the x-axis is shown for confirmed homotypic phase-separating (PS) proteins (blue) and the reference human proteome (black). The percent of each set with PS potential ≥100 is indicated in the figure.

**
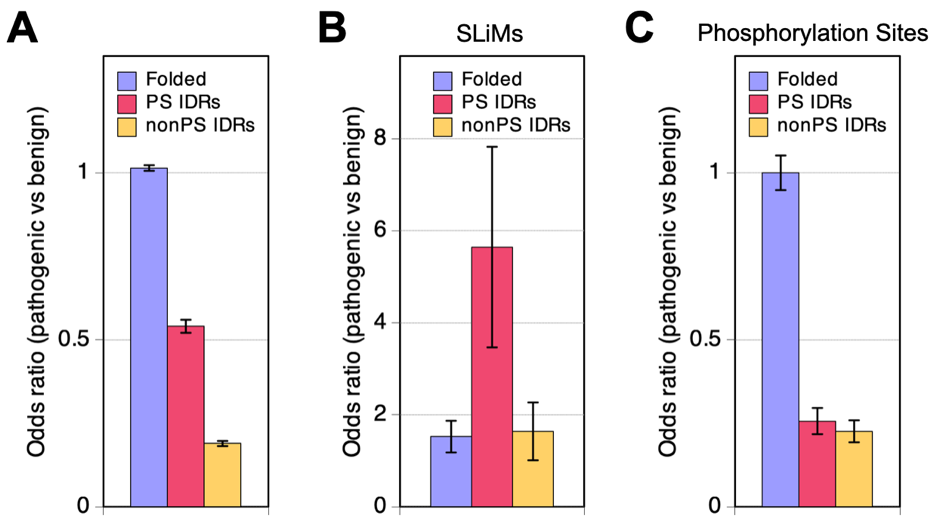
**

**Figure S3.** **Pathogenic versus benign odds ratios for missense mutations.** (**A**) By region class, and for mutations found in (**B**) SLiMs or (**C**) at phosphorylation sites, also by region class. Error bars show the standard error.

**
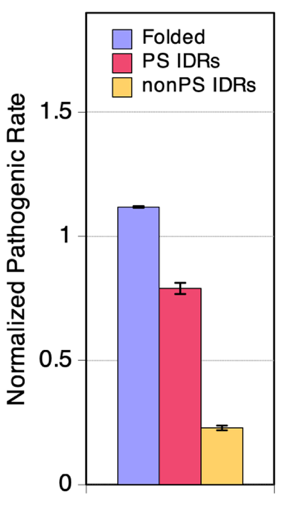
**

**Figure S4.** **Pathogenic missense mutation rate by region class for the natural variants index.** Error bars show the standard error.


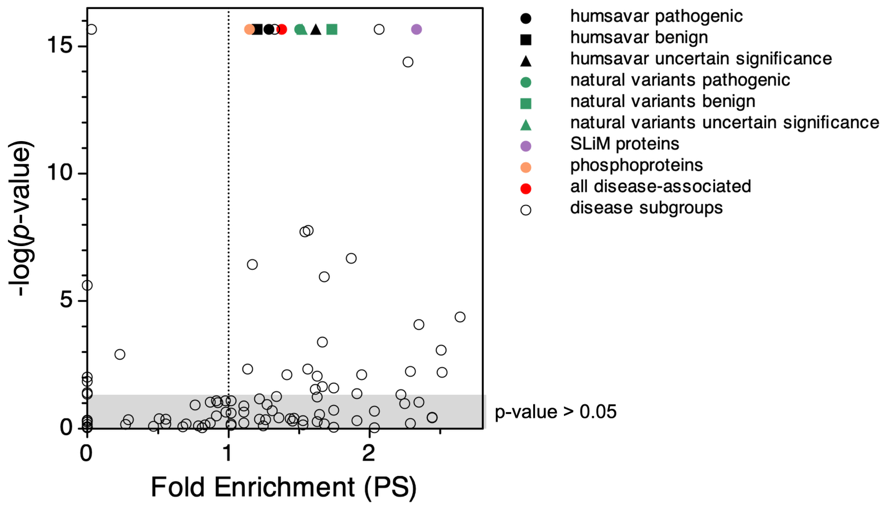


**Figure S5.** **Enrichment for phase separation in protein sets.** Fold enrichment of phase-separating (PS) proteins and one-tail *p*-values from the Mann-Whitney *U*-test (5) were both calculated using the reference human proteome as the comparison set. The black circle, square and triangle show proteins found in the humsavar index with pathogenic, benign, or uncertain clinical significance missense mutations, respectively. The green circle, square, and triangle show proteins found in the natural variants index with pathogenic, benign, or uncertain clinical significance missense mutations, respectively. Purple and salmon circles show proteins with confirmed SLiMs or confirmed phosphorylation sites, respectively. The red circle shows all proteins annotated as disease-associated in UniProt. Open circles show the disease subgroups from Table 3. Protein sets in the grey shaded region did not exhibit statistically significant differences in predicted enrichment for phase separation when compared to the reference human proteome.


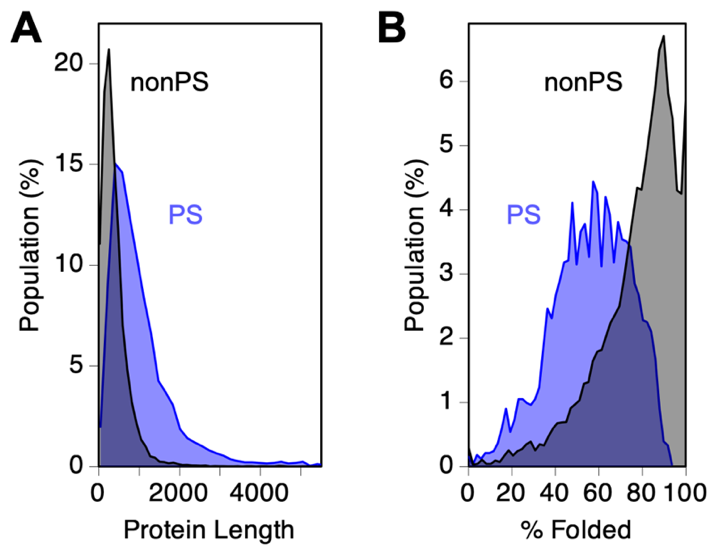


**Figure S6.** **Protein length and percent of residues folded in PS and nonPS proteins.** Populations were calculated using the reference human proteome. PS proteins had sequence-calculated PS potential ≥100; nonPS proteins had sequence-calculated PS potential <100.

**
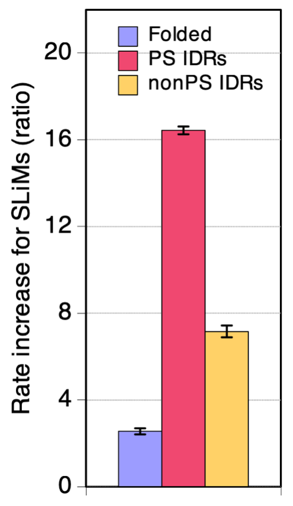
**

**Figure S7.** **Pathogenic missense mutation rate increases in SLiMs by region class.** Shown is the ratio of the pathogenic missense mutation rate for SLiM positions (taken from Figure 2A) divided by the pathogenic rate for both SLiM and nonSLiM positions (taken from Figure 1A) by region class. Values >1 indicate the pathogenic mutation rate was higher for SLiM positions in that region. Error bars show the propagated standard error.


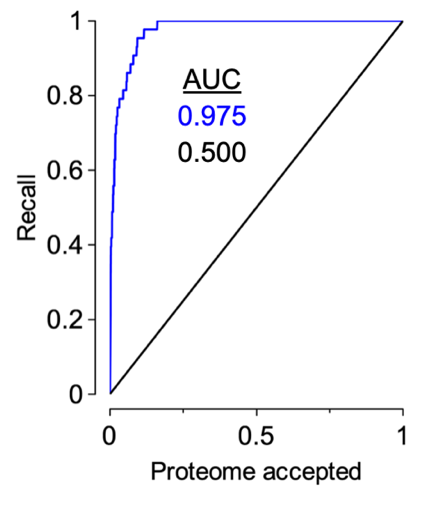


**Figure S8.** **Quantifying differences in predicted PS potential between protein sets.** For a protein set, the percent of proteins with sequence-calculated PS potential equal to or greater than a given value can be computed, as shown in Figure S2 for the reference human proteome and for confirmed homotypic phase-separating (PS) proteins. Percent of set data can be plotted against the human proteome percent of set, as shown here. The human proteome percent of set plotted against itself gives the identity line (black) and yields 0.5 for its area under the curve (AUC). The homotypic PS protein set (blue) plotted against the human reference proteome yields 0.975 for its AUC.


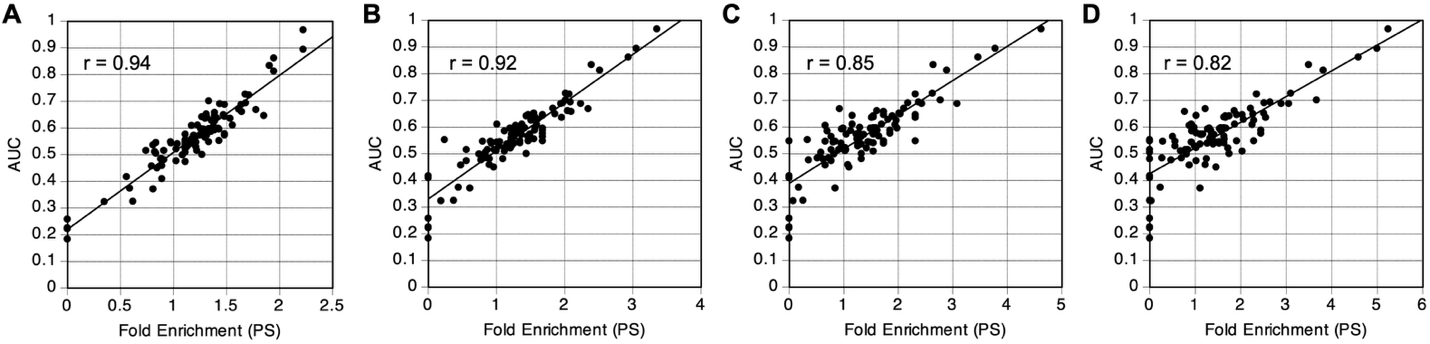


**Figure S9.** **AUC trends with enrichment for phase separation behavior.** Fold enrichment for PS predicted proteins, relative the reference proteome, was calculated using PS potential cutoffs of (**A**) 25, (**B**) 50, (**C**) 75, and (**D**) 100 for the protein sets listed in Table S3. AUC was calculated using the reference human proteome as the comparison set.


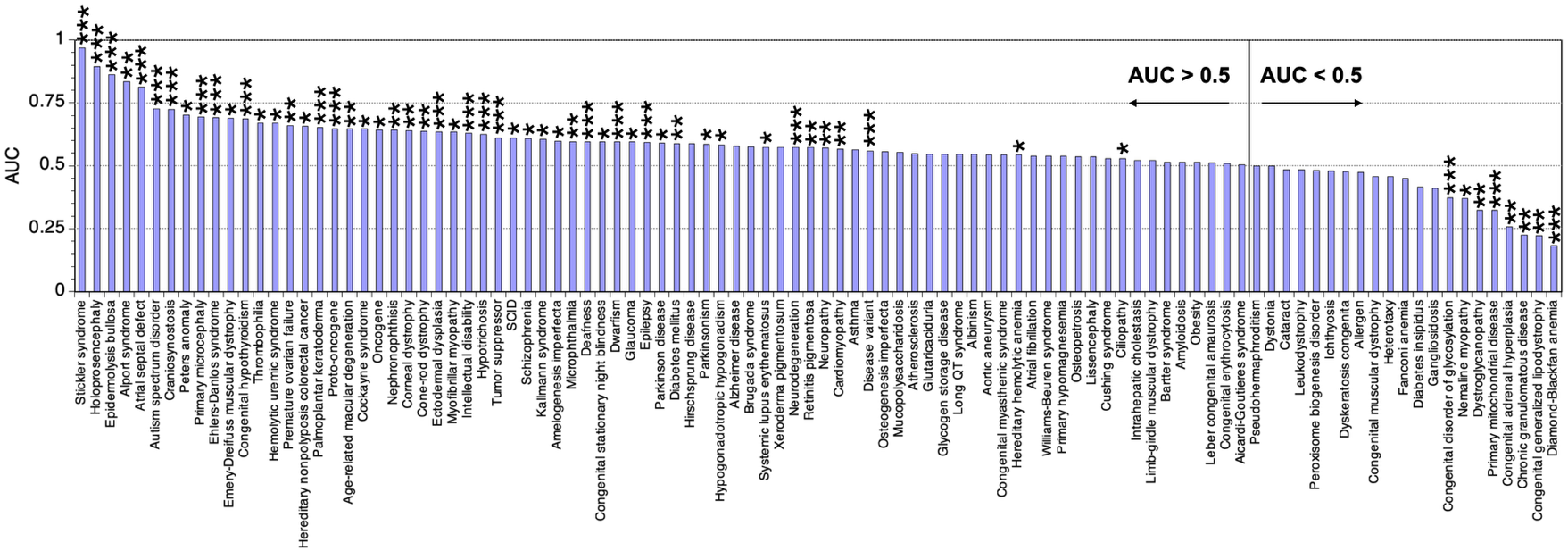


**Figure S10. AUC rank order for protein sets annotated as disease associated.** Curated protein sets confirmed to be associated with human disease were obtained from UniProt (1) and analyzed by ParSe (2). AUC >0.5 predicts enrichment for phase separation behavior. An asterisk marks AUC greater than one standard deviation (𝜎) from the mean AUC of similarly sized random protein sets; two asterisks indicate greater than 2𝜎, and three indicate greater than 3𝜎.


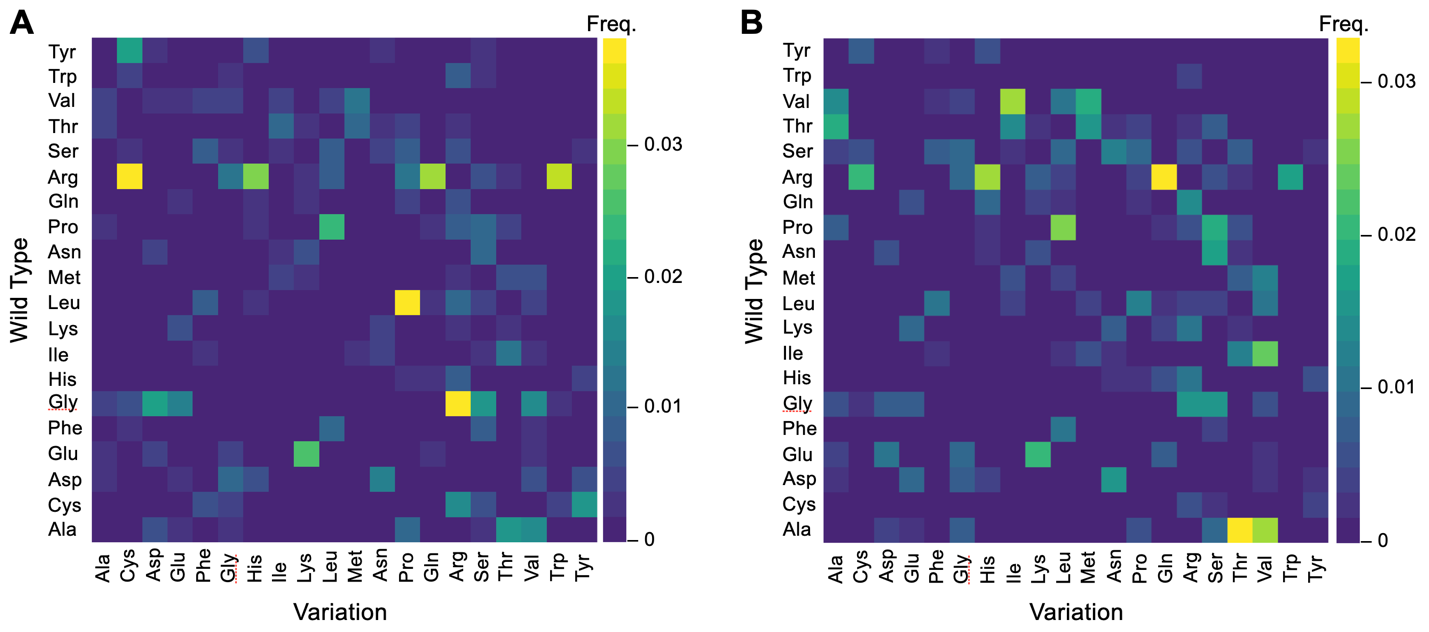


**Figure S11. Frequency of single amino acid missense variation types.** Substitution frequencies (Freq.), representing wild type to variation, were calculated as the number of a type (e.g. Tyr-to-Ala) divided by the total number of single amino acid missense mutations in the humsavar index with (**A**) pathogenic or likely pathogenic and (**B**) benign or likely benign clinical significance. The calculated frequency was colored according to the scale on the right of each plot.


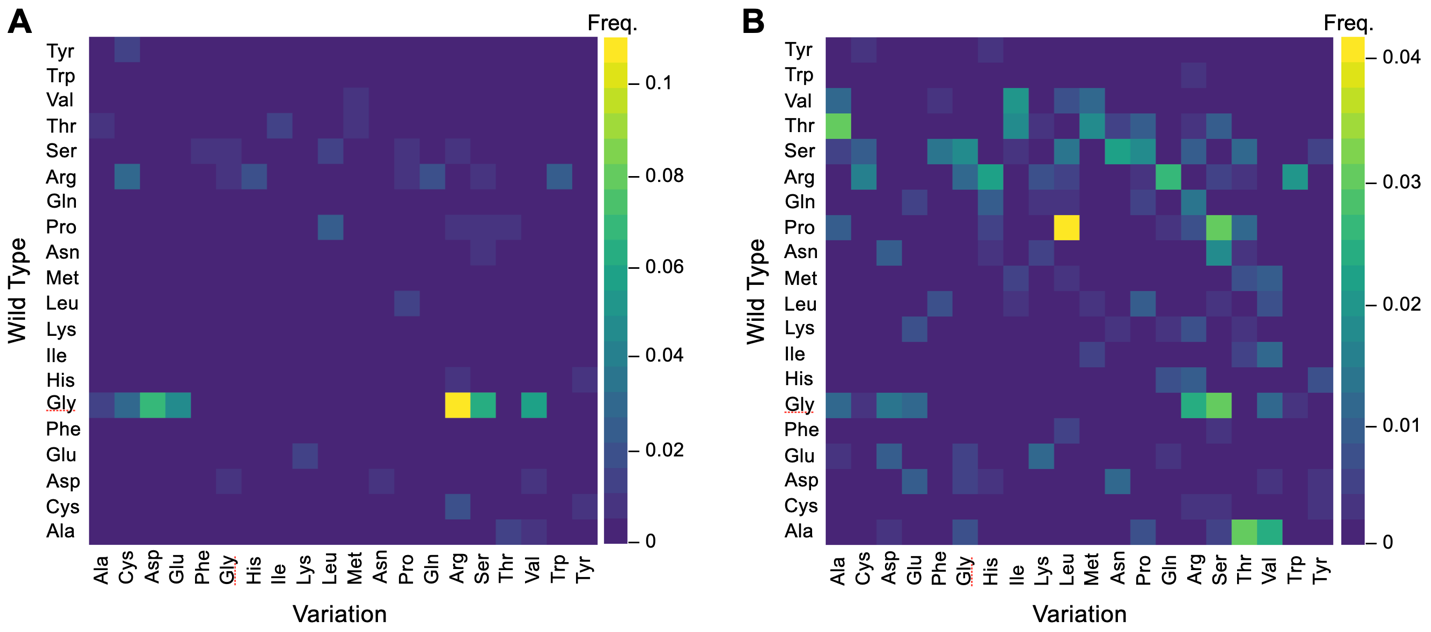


**Figure S12. Frequency of single amino acid missense variation types in PS IDRs.** Substitution frequencies (Freq.) were calculated as the number of a type (e.g. Tyr-to-Ala) found in a PS IDR divided by the total number of single amino acid missense mutations in the humsavar index found in a PS IDR with (**A**) pathogenic or likely pathogenic and (**B**) benign or likely benign clinical significance. The calculated frequency was colored according to the scale on the right of each plot.


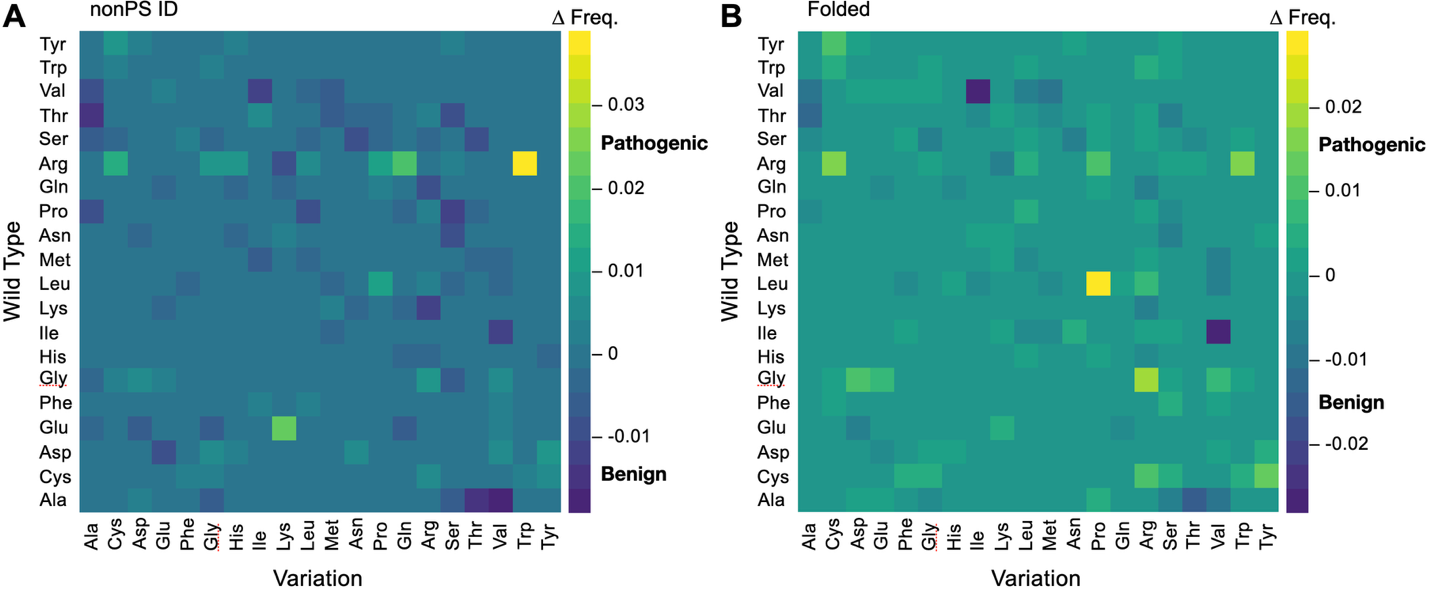


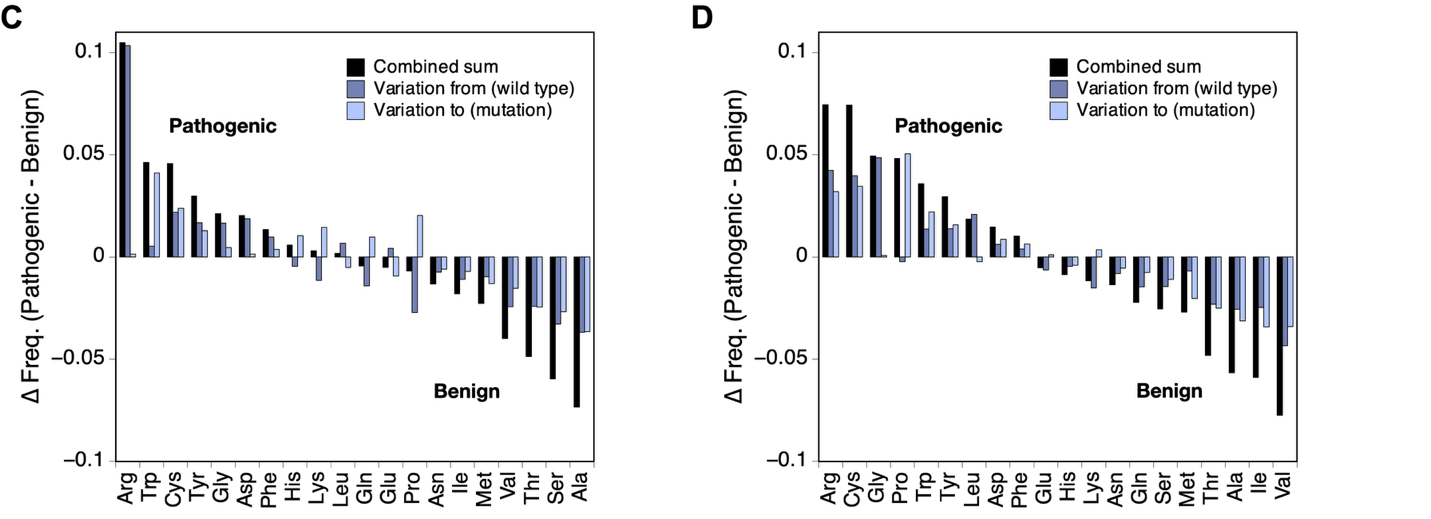


**Figure S13. Pathogenic minus benign difference frequencies for single amino acid missense variation types in nonPS and folded regions.** Difference frequencies (∆ Freq.), colored according to the given scale, were computed from the single amino acid missense variations in the humsavar index for mutations found in regions matching the (**A**) nonPS ID and (**B**) folded classes. Values from (**C**) panel A and (**D**) panel B were summed by wild type amino acid (blue) and mutation amino acid (light blue). The combined sum (black) is the simple addition of the wild type and mutation values.


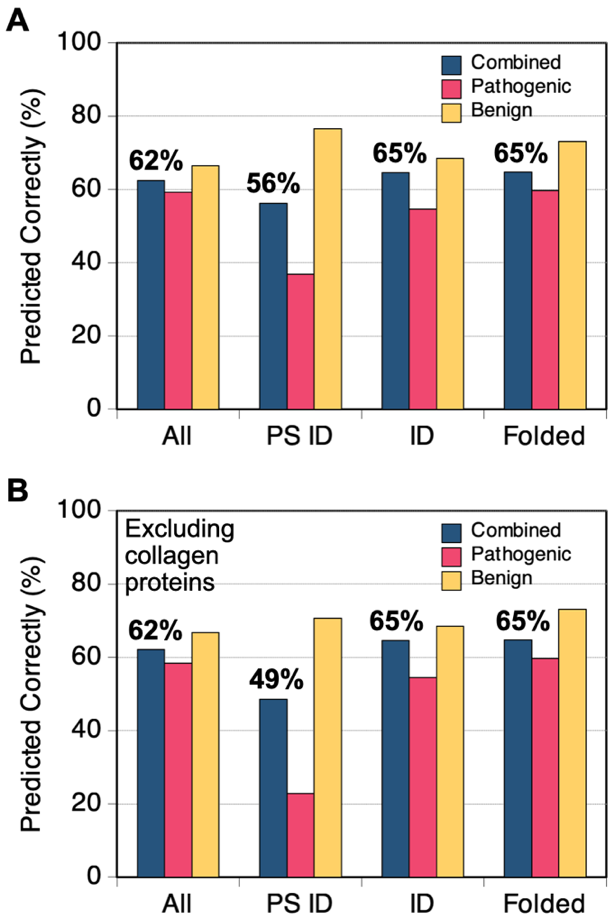


**Figure S14. Predicting clinical significance of missense mutations in the natural variants index.** (**A**) Pathogenic minus benign difference frequencies were calculated for all single amino acid missense mutations in the humsavar index and for the subsets residing in protein regions matching the PS ID, ID (i.e., nonPS ID), or folded classes. Clinical significance was predicted for missense mutations in the natural variants index with known clinical significance where a positive difference frequency predicts pathogenic, and a negative difference frequency predicts benign. Shown is the percent predicted correctly. (**B**) The effect from excluding collagen proteins.

**Supporting References**

1. The UniProt Consortium (2023) UniProt: the Universal Protein Knowledgebase in 2023. *Nucleic Acids Research*. **51**, D523–D531

2. Ibrahim, A. Y., Khaodeuanepheng, N. P., Amarasekara, D. L., Correia, J. J., Lewis, K. A., Fitzkee, N. C., Hough, L. E., and Whitten, S. T. (2023) Intrinsically disordered regions that drive phase separation form a robustly distinct protein class. *J Biol Chem*. **299**, 102801

3. Kumar, M., Michael, S., Alvarado-Valverde, J., Zeke, A., Lazar, T., Glavina, J., Nagy-Kanta, E., Donagh, J. M., Kalman, Z. E., Pascarelli, S., Palopoli, N., Dobson, L., Suarez, C. F., Van Roey, K., Krystkowiak, I., Griffin, J. E., Nagpal, A., Bhardwaj, R., Diella, F., Mészáros, B., Dean, K., Davey, N. E., Pancsa, R., Chemes, L. B., and Gibson, T. J. (2024) ELM—the Eukaryotic Linear Motif resource—2024 update. *Nucleic Acids Research*. **52**, D442–D455

4. Lin, S., Wang, C., Zhou, J., Shi, Y., Ruan, C., Tu, Y., Yao, L., Peng, D., and Xue, Y. (2021) EPSD: a well-annotated data resource of protein phosphorylation sites in eukaryotes. *Brief Bioinform*. **22**, 298–307

5. Mann, H. B., and Whitney, D. R. (1947) On a Test of Whether one of Two Random Variables is Stochastically Larger than the Other. *The Annals of Mathematical Statistics*. **18**, 50–60

6. Patel, A., Lee, H. O., Jawerth, L., Maharana, S., Jahnel, M., Hein, M. Y., Stoynov, S., Mahamid, J., Saha, S., Franzmann, T. M., Pozniakovski, A., Poser, I., Maghelli, N., Royer, L. A., Weigert, M., Myers, E. W., Grill, S., Drechsel, D., Hyman, A. A., and Alberti, S. (2015) A Liquid-to-Solid Phase Transition of the ALS Protein FUS Accelerated by Disease Mutation. *Cell*. **162**, 1066–1077

7. Liu, X., Niu, C., Ren, J., Zhang, J., Xie, X., Zhu, H., Feng, W., and Gong, W. (2013) The RRM domain of human fused in sarcoma protein reveals a non-canonical nucleic acid binding site. *Biochim Biophys Acta*. **1832**, 375–385
